# Affinity Purification Coupled to Stable Isotope Dilution LC-MS/MS Analysis to Discover IgG4 Glycosylation Profiles for Autoimmune Pancreatitis

**DOI:** 10.3390/ijms222111527

**Published:** 2021-10-26

**Authors:** Michael X. Chen, Ho-Hsuan Su, Ching-Ya Shiao, Yu-Ting Chang, Ming-Chu Chang, Chih-Chin Kao, San-Yuan Wang, Hsi-Chang Shih, I-Lin Tsai

**Affiliations:** 1 Department of Pathology and Laboratory Medicine, The University of British Columbia, Victoria, BC V8Z6R5, Canada; Michael.Chen@viha.ca; 2 Division of Medical Sciences, University of Victoria, Victoria, BC V8W2Y2, Canada; 3 Department of Biochemistry and Molecular Cell Biology, School of Medicine, College of Medicine, Taipei Medical University, Taipei 11031, Taiwan; m120109017@tmu.edu.tw (H.-H.S.); smallg507@gmail.com (C.-Y.S.); 4 Department of Internal Medicine, National Taiwan University Hospital, Taipei 100, Taiwan; yutingchang@ntu.edu.tw (Y.-T.C.); mingchuchang@ntu.edu.tw (M.-C.C.); 5 Department of Internal Medicine, College of Medicine, National Taiwan University, Taipei 100, Taiwan; 6 Division of Nephrology, Department of Internal Medicine, Taipei Medical University Hospital, Taipei 11031, Taiwan; 121008@h.tmu.edu.tw; 7 Division of Nephrology, Department of Internal Medicine, School of Medicine, College of Medicine, Taipei Medical University, Taipei 11031, Taiwan; 8 Taipei Medical University-Research Center of Urology and Kidney (TMU-RCUK), Taipei Medical University, Taipei 11031, Taiwan; 9 School of Pharmacy, College of Pharmacy, Taipei Medical University, Taipei 11031, Taiwan; syw@tmu.edu.tw; 10 Master Program in Clinical Genomics and Proteomics, College of Pharmacy, Taipei Medical University, Taipei 11031, Taiwan; 11 Genomics Research Center, Academia Sinica, Taipei 115, Taiwan; hcshih@gate.sinica.edu.tw; 12 Graduate Institute of Medical Sciences, College of Medicine, Taipei Medical University, Taipei 11031, Taiwan; 13 International Ph.D. Program for Cell Therapy and Regeneration Medicine, College of Medicine, Taipei Medical University, Taipei 11031, Taiwan; 14 Pulmonary Research Center, Wan Fang Hospital, Taipei Medical University, Taipei 116, Taiwan

**Keywords:** type 1 autoimmune pancreatitis, IgG4, N-glycosylation, mass spectrometry

## Abstract

Type 1 autoimmune pancreatitis (AIP) is categorized as an IgG4-related disease (IgG4-RD), where a high concentration of plasma IgG4 is one of the common biomarkers among patients. IgG Fc-glycosylation has been reported to be potential biosignatures for diseases. However, human IgG3 and IgG4 Fc-glycopeptides from populations in Asia were found to be isobaric ions when using LC-MS/MS as an analytical tool. In this study, an analytical workflow that coupled affinity purification and stable isotope dilution LC-MS/MS was developed to dissect IgG4 glycosylation profiles for autoimmune pancreatitis. Comparing the IgG4 and glycosylation profiles among healthy controls, patients with pancreatic ductal adenocarcinoma (PDAC), and AIP, the IgG4 glycosylations from the AIP group were found to have more digalactosylation (compared to PDAC) and less monogalactosylation (compared to HC). In addition, higher fucosylation and sialylation profiles were also discovered for the AIP group. The workflow is efficient and selective for IgG4 glycopeptides, and can be used for clinical biosignature discovery.

## 1. Introduction

Autoimmune pancreatitis (AIP) is a distinct form of the chronic fibroinflammatory autoimmune disorder of the pancreas [1]. AIP can be sub-classified into type 1 and type 2 based on the clinical and pathological features. Type 1 AIP, which represents more than 95% of all AIP cases, is a pancreatic manifestation of systemic immunoglobulin G4-related disease (IgG4-RD) characterized by high serum IgG4 concentrations and increased IgG4-positive plasma cells in the tissues [2,3].

Although the serum IgG4 level is commonly used as a biomarker for AIP and IgG4-RD, the measurement of the serum IgG subtype also suffers from several limitations regarding the diagnosis and assessment of disease activity. Approximately 20% of patients with AIP have a serum concentration of IgG4 that is within normal limits [4]. An elevation of serum IgG4 levels is nonspecific to AIP because this happens in a variety of conditions, such as infections and other autoimmune diseases [5]. Elevated serum IgG4 levels have been reported in a significant fraction of patients with pancreatic cancer [6]. Furthermore, a differential diagnosis between AIP and pancreatic ductal adenocarcinoma (PDAC) might be difficult due to the similar clinical symptoms and imaging [7]. Additionally, patients with AIP have a high rate of relapse. The prognostic value of the serum IgG4 level remains limited, as a normal serum concentration IgG4 has been observed in patients with relapsed AIP [8]. To date, there is no simple serum biomarker that can be used as definitive marker for AIP. In a recently published international consensus document [9], research priorities, including the evaluation of additional biomarkers for diagnosis and the monitoring of disease activity, were identified. 

Glycosylation is one of the most abundant post-translational modifications. Immunoglobulin glycosylation controls effector functions of antibodies [10]. It has been shown that altered glycosylation patterns of the immunoglobulin Fc region are associated with rheumatoid arthritis, inflammatory bowel diseases, breast cancer, and gastric cancer [11,12,13,14].

Liquid chromatography–electrospray ionization mass spectrometry (LC–ESI–MS/MS) with affinity purification is a suitable method for IgG glycosylation profiling. This method provides a high resolution and selectivity for many glycan isomers [15]. Our previous work analyzed serum IgG-glycosylation profiles as a differential diagnostic marker [16]. We found that AIP patients had a significantly higher fucosylation of IgG1 and a higher sialylation ratio of IgG subclasses 1, 2, and 4.

The IgG4 Fc-glycosylation profile and its role in AIP or IgG4-RD should be further explored. However, human IgG3 and IgG4 Fc-glycopeptides from Asian populations were found to be isobaric ions, which created analytical challenges by LC-MS/MS [17]. Therefore, the optimization of IgG4 purification, comprehensive target selection for IgG4 glycopeptides, and evaluation of quantitative reliability is crucial for method development and clinical sample analysis. In this study, we developed an analytical workflow that coupled IgG4 affinity purification and stable isotope dilution LC-MS/MS to dissect IgG4 glycosylation profiles for autoimmune pancreatitis.

## 2. Results

### 2.1. Sample Pretreatment Process

#### 2.1.1. Optimization of Volume of Bead Slurry for Human IgG4 Purification

**Figure 1:**
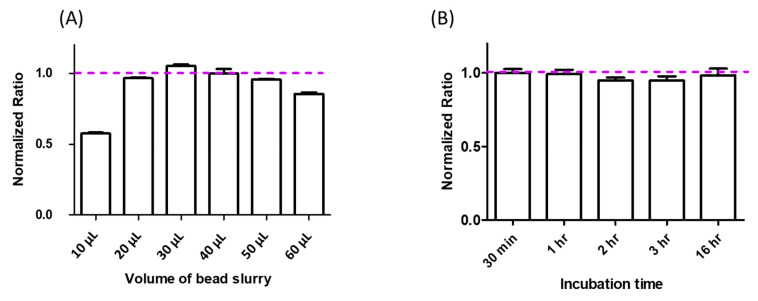
Results of the optimized volume of IgG4 affinity bead slurry and sample incubation time. Peak area of IgG4 surrogate peptide was used for comparison among different conditions. (**A**) Results of the volume of bead slurry used for IgG4 purification. The peak area from the 40 µL condition was used as the denominator for normalization, which was indicated as normalized ratio 1 with a pink dash line. (**B**) Incubation time for IgG4 purification. The peak area from the 30 min condition was used as the denominator for normalization. The standard deviation was calculated from technical replicates (*n* = 3).

To optimize the volume of bead slurry for human IgG4 purification, 10 to 60 µL of bead slurry were conditioned for evaluation. An elevated serum IgG4 from patients with IgG4-RD is anticipated, and a sufficient bead volume and binding capacity were made available. Serum samples from patients with AIP were pooled, and 10 µL of pooling serum was used for each condition to mimic clinical sample with high concentrations of IgG4. The peak area of the IgG4 surrogate peptide was used for comparison, and the results showed that 20 µL of bead slurry was sufficient to purify IgG4. When the bead volume was tested, 20 µL, 30 µL, 40 µL, and 50 µL of bead slurry showed comparable performances, but the signal slightly decreased at the 60 µL condition (Figure 1A). The decrease in peak area might be due to the lower efficiency of on-bead trypsin digestion in the presence of a large bead volume. Considering the variation of the serum IgG4 concentration, 40 µL of human IgG4 affinity bead slurry was chosen in the sample preparation process.

#### 2.1.2. Optimization of Sample Incubation Time

Ten microliters of the AIP pooling serum was used to optimize the suitable time for IgG4 purification. The serum sample was spiked into a microtube with 140 µL of PBS and the conditioned beads. The samples were incubated for 0.5, 1, 2, and 3 h at room temperature and for 16 h at 4 °C by using a bench-top mixer (Figure 1B). By comparing the peak area of the IgG4 surrogate peptide, all of the five conditions showed comparable results. Since 30 min at room temperature was sufficient to purify IgG4, this parameter in the sample preparation process could be tuned and arranged based on the experimental schedule. A longer time of incubation should be conducted at a low temperature to prevent protein degradation.

#### 2.1.3. Wash Step and IgG2 Contamination

**Figure 2:**
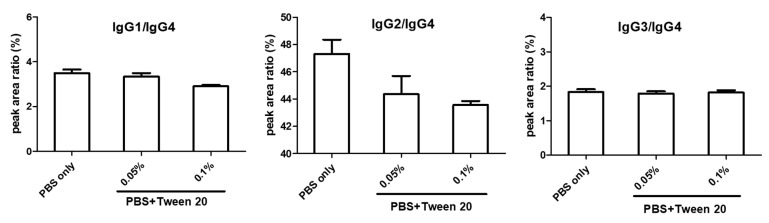
Optimization of wash step after sample incubation. Peak area of surrogate peptides of IgG1, IgG2, and IgG3 were divided by peak area of IgG4 surrogate peptide to investigate the effects of different wash solutions.

The wash step was optimized for affinity purification. Initially, PBS was the only wash solution to reduce the non-specific binding of contaminants. The responses of four surrogate peptides from IgG1, IgG2, IgG3, and IgG4 were monitored by our previous published method [15]. We found that the signals of the IgG2 surrogate peptide were consistently high. The peak area ratios of surrogate peptides for IgG1/IgG4, IgG3/IgG4, and IgG2/IgG4 were performed in Figure 2. The peak area ratios of IgG1/IgG4 and IgG3/IgG4 were less than 4% and 2%, respectively, without adding Tween 20 in the wash solution. In contrast, the peak area ratios of IgG2/IgG4 were around 45%. Although the presence of IgG2 in the purified sample could be easily distinguished by mass spectrometry, we tried to add detergent in the wash step to improve the purification results. However, the peak area ratio of IgG2/IgG4 only decreased slightly with the presence of 0.05% or 0.1% of Tween 20 in the PBS wash solution.

We designed an additional experiment to investigate the co-purification of IgG2 by using CaptureSelect IgG4 affinity beads. Two samples were prepared by mixing IgG2, chicken serum, and PBS to mimic serum samples with 4.8 μg/μL of human IgG2. In one of the samples, we added IgG4, which mimicked the serum sample with both 4.8 μg/μL of human IgG2 and 0.6 μg/μL of IgG4. After incubating the samples at 37 °C for 1 h, they were processed with the developed sample preparation method prior to UHPLC-MS/MS analysis. The results showed that, when there was no IgG4 in the sample, a low amount of IgG2 was detected due to non-specific binding on the beads. Furthermore, the peak area of IgG2 was increased by approximately three times with the presence of IgG4 (data not shown). Based on the results, we assumed that there might be a molecular interaction of IgG4 and IgG2, which enhanced the co-purification of IgG2 from the sample. However, IgG2 affinity beads are not currently commercially available for further investigations. 

#### 2.1.4. Optimization of on-Bead Digestion Time

**Figure 3:**
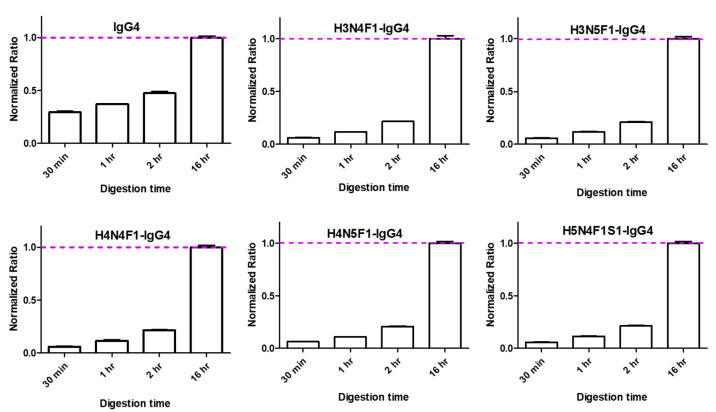
Results of optimized on-bead digestion time. Digestion times from 30 min to 16 h were conducted for comparison. The peak areas of the IgG4 surrogate peptide and selected IgG4 glycopeptides were performed. Peak area from the 16 h condition was used as the denominator for normalization and was indicated with a pink dash line. The standard deviation was calculated from technical replicates (*n* = 3).

In this study, on-bead trypsin digestion was adopted without the need for acidic protein elution or sample matrix replacement prior to digestion. After reducing the disulfide bonds with DTT and alkylating the cysteines with IAA, 5 µL of trypsin (200 ng/µL) was added to each sample. Four conditions, including 0.5, 1, 2, and 16 h at 37 °C (300 rpm), were tested for comparison. In Figure 3, results showed that 16 h of trypsin digestion obtained the highest peak areas of the IgG4 surrogate peptide and glycopeptides. We also observed that peak areas of glycopeptides from samples with 16 h of digestion were approximately four times higher than those with 2 h of digestion. In contrast, the fold change was only two times for the IgG4 surrogate peptide between 16 and 2 h of digestion. This phenomenon was consistent with our previous work for the total IgG Fc glycopeptides analysis, which indicated that glycopeptides take a longer time to be well digested due to the steric hindrance [15,18]. 

### 2.2. UHPLC-MS/MS Analysis and the General Workflow

Reversed-phase liquid chromatography is an efficient and effective technique for the separation of glycopeptides and peptides. The triple quadrupole mass spectrometer (TQMS) provides sensitivity and selectivity, and has been used for biomolecules detection from complex samples. In this study, UHPLC-MS/MS was chosen as the analytical platform. Method-specific parameters and performances were evaluated.

#### 2.2.1. UHPLC-MS/MS Condition

A narrow-bore column packed with the C18 stationary phase was used for glycopeptides/peptides separation. The column length is 5 cm and the internal diameter is 2.1 mm. The mobile phase comprised water and acetonitrile, with the addition of formic acid to a final concentration of 0.1%, and gradient elution was conducted during the analysis. The presence of an acidic additive facilitates the ionization efficiency of target analytes in the electrospray ionization source. Detailed conditions of UHPLC can be found in Section 4.3. In order to investigate N-glycoforms on the IgG4 Fc region, we finally targeted fifteen IgG4 glycopeptides, which showed a reliable repeatability and accuracy for biomarker discovery. Analytes were detected by TQMS with a multiple reaction monitoring (MRM) detection mode. The m/z of precursor ions for glycopeptides were calculated by using Glyco Mass Calculator [19], and the two common fragment ions, m/z 204.1 and 366.1, represented N-acetylhexose (HexNAc) and hexose-HexNAc (Hex-HexNAc), respectively. Parameters for IgG4 and internal standard (IS) peptides were firstly predicted by Skyline software and later selected according to the experimental results. MRM parameters of the 15 IgG4 glycopeptides, IgG4 surrogate peptide, and stable-isotope-labeled internal standard (SIL-IS) were summarized in Table 1. The compositions of the N-glycosylation were indicated as: H, hexose; N, N-acetylglucosamine; F, fucose; S, sialic acid. Mass spectra of the IgG4 surrogate peptide and five representative IgG4 glycopeptides were provided in Supplementary Figure S1. The IgG4 glycopeptides were clustered around 2.40 min, and the retention time of the IgG4 peptide was at 4.78 min.

**Figure 4:**
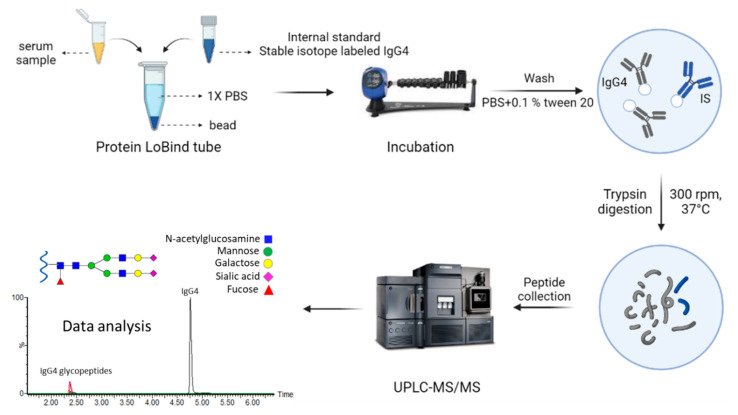
Workflow of affinity purification coupled to UHPLC-MS/MS analysis for IgG4 and glycopeptides investigation.

Since trypsin digestion was conducted during the sample preparation, we selected a SIL-IgG4 as the internal standard, which was added during IgG4 purification. The workflow started from affinity purification, where 10μL of the serum and 10 μL of SIL-IS IgG4 (0.2 μg μL^−1^) were incubated with conditioned affinity beads. After sample incubation, the beads were washed with PBS and PBS with 0.1% Tween 20, and the on-bead digestion was conducted by adding sufficient trypsin enzyme (37 ℃). The enzymatic reaction was stopped after 16 h by adding formic acid, and the tryptic peptides in the supernatant were carefully collected. The supernatant was further diluted with 0.1% formic acid four times prior to UHPLC-MS/MS analysis (Figure 4).

#### 2.2.2. Performance of the Analytical Method

It was challenging to generate calibration curves for IgG4-glycopeptides with exact concentrations due to the lack of glycopeptide standards. Instead, we generated calibration curves for glycopeptides by spiking different IgG4 concentrations. The responses of the IgG4 glycopeptides were used as y-values, and the spiked IgG4 concentrations were used as x-values. In Table 2, we adopted linear equations (y = ax + b) for calibrator fitting. The r values were in the range from 0.917 to 0.999. The calibration curves were not used to report the exact concentrations of glycopeptides, but to provide the information that the relative quantification and comparison of glycopeptides among different samples or groups were reliable. The lower limit of quantification (LLOQ) was determined based on the precision and accuracy evaluation. Eight of the fifteen IgG4-glycopeptides showed lower signal intensities, which could not be determined accurately at low IgG4 spiked concentrations. In Supplementary Table S1, we also summarized the results of the repeatability for both the signal responses (peak area ratio) and retention times of analytes. The relative standard deviation (RSD) of intra-batch and inter-batch precisions for signal responses was less than 33.5 and 36.4%, respectively. The percentage recoveries of intra-batch and inter-batch accuracies for signal responses were in the range from 72.3~105.4% and 76.9~112.0%, respectively. The RSD values calculated for the retention times were less than 0.57% RSD. We also evaluated IgG4 stabilities at three different concentrations in a −20 ℃ freezer for 7 and 15 days, and the recoveries were 98.2~104.1%. The stabilities of IgG4 after three freeze and thaw cycles were 85.3~93.7%, and the stabilities of the IgG4 surrogate peptide in the 6 ℃ autosampler after 24 h were 89.4~96.0% ( Supplementary Table S2).

### 2.3. Application to Clinical Samples

**Figure 5:**
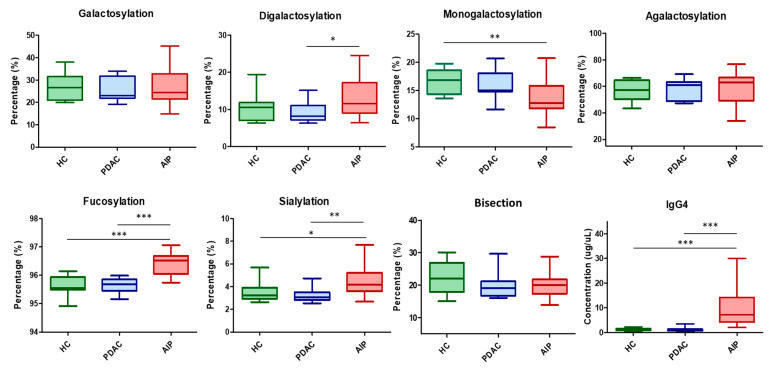
IgG4 showed different glycosylation patterns between groups. HC, healthy controls; PDAC, pancreatic ductal adenocarcinoma; AIP, autoimmune pancreatitis. * represents *p* < 0.05; ** represents *p* <0.01; *** represents *p* <0.0001 for Mann–Whitney statistical analysis.

We have applied the developed workflow to 45 subjects that were grouped into a healthy control (HC), patients with pancreatic ductal adenocarcinoma (PDAC), and patients with autoimmune pancreatitis (AIP). IgG4 concentrations determined by UHPLC-MS/MS were statistically higher in the AIP group (Figure 5). After comparing the glycosylation patterns among the three groups, we found that the IgG4 from patients with AIP has more digalactosylation (compared to PDAC) and less monogalactosylation (compared to HC). However, there were no significant differences among the groups when we evaluated the galactosylation, agalactosylation, and bisection profiles. In addition, we also found that the IgG4 from AIP patients has higher fucosylation and sialylation profiles (Figure 5). The statistical results of the 15 IgG4 glycopeptides were provided in Supplementary Figure S2.

## 3. Discussion

Compared to the methods in our previous publications, the current workflow can determine more IgG4 glycopeptides. Sample preparation conditions, such as the volume of bead slurry, incubation time, wash steps, and trypsin digestion time, were carefully optimized for clinical application. A stable-isotope-labeled IgG4 IS was added to compensate for the variations during sample preparation and UHPLC-MS/MS analysis.

Konno N et al. have used a sequential purification method for IgG4 glycosylation study in which Melon gel was firstly adopted for total IgG purification followed by IgG4 affinity beads. In their study, purification was conducted in columns that were packed with IgG4 affinity beads, and 23 glycans released from IgG4 were analyzed by using MALDI-TOF MS [20]. In contrast, we used IgG4 affinity beads to purify human IgG4 directly without purifying the total IgG. Purification and sample pretreatment were all conducted in the same microtube, which was convenient and cost-effective. Although we only targeted 15 IgG4 glycopeptides in clinical samples, they were carefully selected based on precision and accuracy studies to provide a reliable glycosylation profile among different groups. In addition, the affinity purification used in this study eliminated isobaric interferences in IgG3 and IgG4 glycopeptides. The workflow is efficient and accurate for clinical applications. Additionally, this pipeline can be adopted for the analysis of other glycoproteins.

Hu C et al. used a lectin array to investigate the IgG4 glycosylation profiles for IgG4-RD. In their study, IgG4 from IgG4-RD patients showed distinct patterns compared to disease and healthy controls, and the levels of mannose and GlcNAc were highlighted as being worthy for further investigation [21]. Culver E et al. also investigated IgG Fc-glycosylation profiles for IgG4-RD by using nanoLC-MS. Comparing to healthy controls, the IgG4 from patients with IgG4-RD showed less galactosylation and more fucosylation in their study [22]. Konno N et al. specifically purified human IgG4 for a detailed IgG4 glycosylation study for IgG4-RD. It was reported that agalactosylation (G0) and fucosylation were greater in the IgG4-RD group compared to the healthy controls [20]. In our previously published work, IgG4 sialylation was found to be greater in the AIP group, but the fucosylation percentage was only found to have a significant difference for the IgG1 subclass [16]. In this study, we confirmed that both the sialylation and fucosylation of IgG4 were greater for the AIP group. Although there was no statistical difference for galactosylation between the HC and AIP groups, the IgG4 from AIP patients has a lower monogalactosylation (G1) percentage compared to HC, and more digalactosylation compared to the PDAC group. The finding of the bisection profile was similar to other studies, where there were no significant differences among the groups. In Supplementary Figure S2, six IgG4 glycopeptides with sialic acid were greater in the AIP group, which resulted in the higher sialylation profile (Figure 5). Ten IgG4 glycopeptides with fucose were also found to be greater in the AIP group. In contrast, those without fucose, such as H3N5-IgG4 and H4N5-IgG4, had lower normalized responses in the AIP group. We also observed that five glycopeptides with digalactoses were all higher in the AIP group. However, the trends of glycopeptides with monogalactose or bisecting glycan are lacking in consistency. These results were associated with bisection, galactosylation, and agalactosylation profiles, where there were no significant differences among the groups.

There is evidence that endogenous IgG with more fucosylation and sialylation has anti-inflammation biological functions [23]. However, effector analyses or biological studies regarding IgG4 were mostly focused on IgG4 monoclonal antibodies (mAbs) [24]. For example, Gong Q et al. found that the afucosylated percentage of IgG4 monoclonal antibodies was related to their activity of antibody-dependent cell-mediated cytotoxicity (ADCC) [25]. The association of endogenous IgG4 glycosylation to the downstream effector functions should be the focus of future research.

One of the limitations of this study is the small sample size; therefore, each group was only assigned 15 subjects. Although the purification and sample preparation steps are efficient, we might miss some of the minor glycans on the IgG4 Fc region, which were detected by the glycomics method [20].

## 4. Materials and Methods

### 4.1. Standards and Reagents

Protein standards of natural human IgG2 and IgG4 were purchased from Abcam (Cambridge, MA, USA). The protein internal standard used in this study was SILu™MAB K4 Stable-Isotope-labeled universal monoclonal antibody, which was bought from Sigma-Aldrich (St. Louis, MO, USA). Ammonium bicarbonate (ABC), formic acid, dithiothreitol (DTT), and iodoacetamide (IAA) were bought from Sigma-Aldrich (St. Louis, MO, USA). Chicken serum and CaptureSelect IgG4 (Hu) affinity matrix were purchased from Thermo Fisher Scientific (Sunnyvale, CA, USA). Trypsin enzyme was purchased from Promega (Madison, WI, USA). Acetonitrile (ACN) was obtained from J.T. Baker (MS grade, Phillipsburg, NJ, USA). Phosphate-buffered saline (10×) was purchased from VWR International LLC (West Chester, PA, USA).

### 4.2. Sample Preparation Process

Forty microliters of CaptureSelect IgG4 (Hu) affinity matrix were conditioned with PBS. After conditioning twice, 10 μL of human serum was mixed with 140 μL of PBS and affinity beads. In addition, 10 μL of SIL-IS IgG4 (0.2 μg μL^−1^) was added to each sample solution. The IgG4 purification was conducted at room temperature for 30 min with a desktop mixer. After incubation, the supernatant was removed, and the beads were washed with PBS once, 0.1% Tween 20 in PBS twice, followed by PBS again to decrease non-specific binding. Fifty microliters of 50 mM ABC were added to the microtube, and 1 μL of 550 mM DTT was added to the solution to disrupt the disulfide bonds (56 °C for 45 min). The solution was then treated with 2 μL of 450 mM IAA as an alkylating reagent at room temperature for 45 min in the dark. For protein digestion, 10 μL of trypsin (200 ng μL^−1^) was spiked and the digestion was conducted at 37 °C for 16 h (300 rpm). At the end of on-bead digestion, formic acid was added to stop the tryptic digestion. The supernatant was collected after centrifuging the sample and was further diluted with 0.1% formic acid 4 times before UHPLC-MS/MS analysis.

### 4.3. UHPLC-MS/MS Analysis

The UHPLC-MS/MS analysis was performed on Waters ACQUITY UPLC system and the Xevo TQ-XS triple quadruple mass spectrometry (Waters Corporation, Milford, MA, USA). The separation column used in this study was Core-Shell C18 Kinetex column (50 mm × 2.1 mm, 2.6 µm; Phenomenex, Torrance, CA, USA). The mobile phase was composed of 0.1% formic acid in water (solvent A) and 0.1% formic acid in ACN (solvent B). Column temperature was set at 40 °C and the flow rate of mobile phase was set at 0.3 mL min^−1^. Gradient elution was used with the following setting: 2% solvent B for 0–0.5 min; 2–50% solvent B for 0.5–8 min; 50% solvent B for 8–8.5 min; 50–100% solvent B for 8.5–9 min; 100% solvent B for 9–11 min. The mobile phase was restored to 2% B at 11.5 min and maintained at 2% for additional 1.5 min to achieve column equilibration. Five microliters of sample were introduced for analysis. The parameters of electrospray ionization positive mode are listed as follows: capillary voltage, 2.5 kV; source offset, 30 V; source temperature, 150 °C; desolvation temperature, 450 °C; collision gas flow, 0.15 mL min^−1^; nebulizer gas flow, 7 bar.

### 4.4. Performance of the UHPLC-MS/MS Method

Calibration curves were generated by adding a serial concentration of IgG4 standards into chicken serum, as indicated in Table 2. The samples were prepared and analyzed by the developed method, and the peak area ratios of target analyte to the IS were used as *Y*-axis to generate calibration curves. Linear regression was used to construct the equation for each analyte. Precision and accuracy were evaluated at different IgG4 spiked concentrations. The intra-batch and inter-batch repeatabilities were calculated and performed as % RSD for both retention times and peak area ratios. Accuracies were calculated as the percentage of recovery by comparing the experimental concentrations to the theoretical ones. For glycopeptides, spiked protein concentrations were used for accuracy evaluation as well. Stabilities of IgG4 were evaluated by comparing the signals from samples kept at −20℃ for 7 and 15 days to the fresh prepared one. The stability of surrogate peptide in autosampler was evaluated at 6℃ for 24 h.

### 4.5. Analysis of Clinical Samples

Clinical samples were collected from patients with AIP, PDAC, and healthy controls to investigate the differences in IgG4 subclass concentrations and the Fc-glycosylation profiles (*n* = 15/group). This study was approved by the research ethics committee of National Taiwan University Hospital (201301048RIND) and Taipei Medical University–Joint Institutional Review Board (N201704064). The signed informed consent forms were received from all subjects.

### 4.6. Data analysis and Glycosylation Profiling

UHPLC-MS/MS data were processed with MassLynx (vers. 4.2, Waters Corporation, Milford, MA, USA). Before calculating the glycosylation profiles (%), the response of each glycopeptide was divided by the sum of responses from all glycopeptides to obtain its percentage value. Different glycosylation profiles were then calculated based on the following Equations (1)–(7). To compare the 15 glycopeptides among the three groups, the responses of glycopeptides were normalized with their respective IgG4 response to prevent the effect of different protein amounts from samples. Statistical analysis was conducted in GraphPad Prism (vers. 5.01, San Diego, CA, USA).

Galactosylation: (H4N4F1-IgG4 + H4N4F1S1-IgG4 + H4N5-IgG4 + H4N5F1-IgG4 + H4N5F1S1-IgG4 + H4N5S1-IgG4) × 0.5 + (H5N4F1-IgG4 + H5N4F1S1-IgG4 + H5N4S2-IgG4 + H5N5F1-IgG4 + H5N5S1-IgG4)(1)

Monogalactosylation (G1): (H4N4F1-IgG4 + H4N4F1S1-IgG4 + H4N5-IgG4 + H4N5F1-IgG4 + H4N5F1S1-IgG4 + H4N5S1-IgG4) × 0.5(2)

Digalactosylation (G2): H5N4F1-IgG4 + H5N4F1S1-IgG4 + H5N4S2-IgG4 + H5N5F1-IgG4 + H5N5S1-IgG4(3)

Agalactosylation (G0): H3N3F1-IgG4 + H3N4F1-IgG4 + H3N5-IgG4 + H3N5F1-IgG4(4)

Bisection: H3N5-IgG4 + H3N5F1-IgG4 + H4N5-IgG4 + H4N5F1-IgG4 + H4N5F1S1-IgG4 + H4N5S1-IgG4 + H5N5F1-IgG4 + H5N5S1-IgG4(5)

Fucosylation: H3N3F1-IgG4 + H3N4F1-IgG4 + H3N5F1-IgG4 + H4N4F1-IgG4 + H4N4F1S1-IgG4 + H4N5F1-IgG4 + H4N5F1S1-IgG4 + H5N4F1-IgG4 + H5N4F1S1-IgG + H5N5F1-IgG4(6)

Sialylation: (H4N4F1S1-IgG4 + H4N5F1S1-IgG4 + H4N5S1-IgG4 + H5N4F1S1-IgG4 + H5N5S1-IgG4) × 0.5 + H5N4S2-IgG4(7)

## 5. Conclusions

In this study, we have carefully optimized sample preparation for IgG4 purification and on-bead tryptic digestion. SIL-IgG4 IS was used to achieve a more accurate and precise analysis. The IgG4 serum concentration and the 15 IgG4 glycopeptides can be determined by using the developed UHPLC-MS/MS method. We employed this workflow to generate IgG4 glycosylation profiles from healthy controls, patients with PDAC, and patients with AIP. Higher fucosylation and sialylation profiles were found in the AIP group. In addition, AIP was found to have more digalactosylation compared to the PDAC group. Our workflow provides an efficient way for IgG4 Fc-glycosylation profiling. It has the potential to be applied to other biological investigations.

## Figures and Tables

**Table 1:** MRM transitions and parameters for the IgG4 surrogate peptide, glycopeptides, and the internal standard.

Compound Name	RT (min)	Precursor (m/z)	Product (m/z)	Cone (V)	Collision (eV)
IgG4: TTPPVLDSDGSFFLYSR	4.77	951.5	850.4/1293.6	35	34
H3N3F1-IgG4 ^a^	2.38	805.7	204.1/366.1	35	25
H3N4F1-IgG4	2.38	873.4	204.1/366.1	35	25
H3N5-IgG4	2.40	892.4	204.1/366.1	35	25
H3N5F1-IgG4	2.39	941.0	204.1/366.1	35	25
H4N4F1-IgG4	2.36	927.4	204.1/366.1	35	25
H4N4F1S1-IgG4	2.46	1024.4	204.1/366.1	35	25
H4N5-IgG4	2.40	946.4	204.1/366.1	35	25
H4N5F1-IgG4	2.38	995.1	204.1/366.1	35	25
H4N5F1S1-IgG4	2.48	1092.1	204.1/366.1	35	25
H4N5S1-IgG4	2.43	1043.4	204.1/366.1	35	25
H5N4F1-IgG4	2.35	981.4	204.1/366.1	35	25
H5N4F1S1-IgG4	2.44	1078.4	204.1/366.1	35	25
H5N4S2-IgG4	2.38	1126.8	204.1/366.1	35	25
H5N5F1-IgG4	2.37	1049.1	204.1/366.1	35	25
H5N5S1-IgG4	2.44	1097.4	204.1/366.1	35	25
IS: TTPPVLDSDGSFFLYSR{^13^C6,^15^N4}	4.78	956.5	806.9/855.4/303.6	35	34

^a^ The peptide sequence for IgG4 glycopeptide: EEQFNSTYR. H, hexose; N, N-acetylglucosamine; F, fucose; S, sialic acid.

**Table 2:** The calibration curves for IgG4 and 15 IgG4 glycopeptides.

Analytes	Calibration Curves (y = ax + b)			
	Spiked Protein Concentration(μg μL^−1^)	a	b	r
IgG4	0.14~8.80	7.392	0.0836	0.999
H3N3F1-IgG4	0.14~8.80	0.017	−0.0001	0.992
H3N4F1-IgG4	0.14~8.80	0.813	0.0392	0.995
H3N5-IgG4 ^a^	0.28~8.80	0.024	0.0063	0.966
H3N5F1-IgG4	0.14~8.80	0.113	0.0024	0.995
H4N4F1-IgG4	0.14~8.80	0.577	0.0228	0.996
H4N4F1S1-IgG4	0.14~8.80	0.051	0.0002	0.995
H4N5-IgG4 ^a^	0.28~8.80	0.019	0.0041	0.968
H4N5F1-IgG4	0.14~8.80	0.098	0.0020	0.995
H4N5F1S1-IgG4 ^a^	0.55~8.80	0.004	−0.0007	0.979
H4N5S1-IgG4 ^a^	0.55~8.80	0.002	−0.0003	0.917
H5N4F1-IgG4	0.14~8.80	0.071	0.0008	0.995
H5N4F1S1-IgG4 ^a^	0.28~8.80	0.029	−0.0003	0.994
H5N4S2-IgG4 ^a^	0.55~8.80	0.003	−0.0005	0.980
H5N5F1-IgG4 ^a^	0.55~8.80	0.007	−0.0009	0.984
H5N5S1-IgG4 ^a^	0.55~8.80	0.001	−0.0003	0.948

^a^ Analytes with smaller signals that could not be determined at 0.14 μg μL^−1^ spiked IgG4 concentrations.

## Data Availability

The data that have been used to support the findings of this study are available from the corresponding author upon request.

## Supplementary Materials

The following are available online at https://www.mdpi.com/article/10.3390/ijms222111527/s1.
