# Supplementary material for: Affinity Purification Coupled to Stable Isotope Dilution LC-MS/MS Analysis to Discover IgG4 Glycosylation Profiles for Autoimmune Pancreatitis
Source: Int J Mol Sci. 2021 Oct 26;22(21):11527. doi: 10.3390/ijms222111527 (PMC8584136; doi:10.3390/ijms222111527)
Supplement: Supplementary file 1 [file ijms-22-11527-s001.zip › Supplementary Figure S1_MS spectra.pdf]

Supplementary Figure S1. Mass spectra of (A) IgG4 surrogate peptide, and (B) representative IgG4 glycopeptides collected by the product ion scan mode.

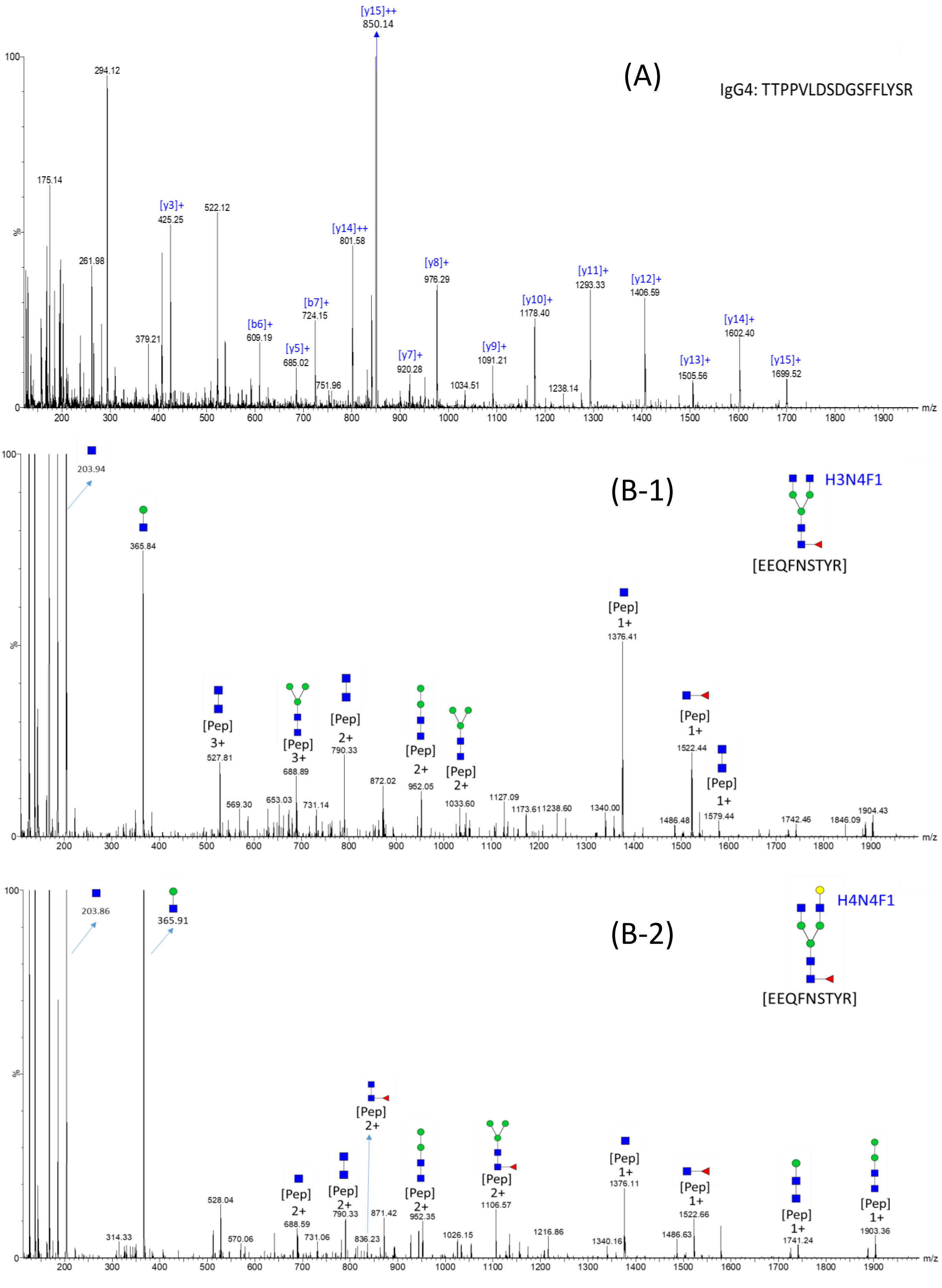

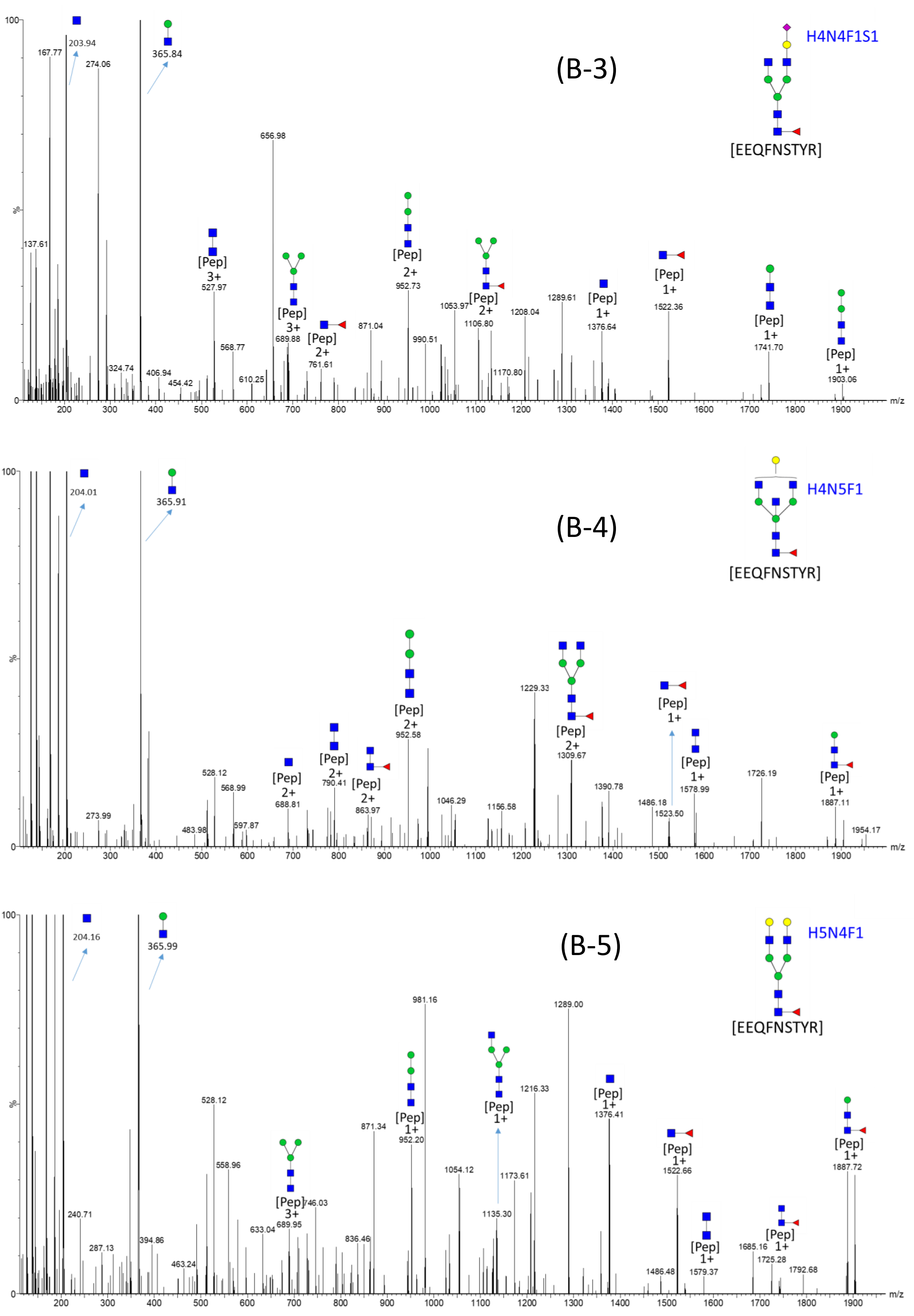
(B-3)
H4N4F1S1
[EEQFNSTYR]
100
%
0
m/z
203.94
365.84
167.77
274.06
137.61
656.98
324.74
406.94
454.42
[Pep] 3+ 527.97
568.77
610.25
[Pep] 3+ 689.88
[Pep] 2+ 761.61
871.04
[Pep] 2+ 952.73
990.51
1053.97
[Pep] 2+ 1106.80
1170.80
1208.04
1289.61
[Pep] 1+ 1376.64
[Pep] 1+ 1522.36
[Pep] 1+ 1741.70
[Pep] 1+ 1903.06
(B-4)
H4N5F1
[EEQFNSTYR]
100
%
0
m/z
204.01
365.91
273.99
483.98
528.12
568.99
597.87
[Pep] 2+ 688.81
[Pep] 2+ 790.41
[Pep] 2+ 863.97
[Pep] 2+ 952.58
1046.29
1156.58
1229.33
[Pep] 2+ 1309.67
1390.78
1486.18
[Pep] 1+ 1523.50
[Pep] 1+ 1578.99
1726.19
[Pep] 1+ 1887.11
1954.17
(B-5)
H5N4F1
[EEQFNSTYR]
100
%
0
m/z
204.16
365.99
240.71
287.13
394.86
463.24
528.12
558.96
633.04
[Pep] 3+ 689.95
746.03
836.46
871.34
[Pep] 1+ 952.20
981.16
1054.12
[Pep] 1+ 1135.30
1173.61
1216.33
1289.00
[Pep] 1+ 1376.41
1486.48
[Pep] 1+ 1522.66
[Pep] 1+ 1579.37
1685.16
[Pep] 1+ 1725.28
1792.68
[Pep] 1+ 1887.72
