# Supplementary material for: Affinity Purification Coupled to Stable Isotope Dilution LC-MS/MS Analysis to Discover IgG4 Glycosylation Profiles for Autoimmune Pancreatitis
Source: Int J Mol Sci. 2021 Oct 26;22(21):11527. doi: 10.3390/ijms222111527 (PMC8584136; doi:10.3390/ijms222111527)

Supplementary Figure S2. Statistical results of 15 IgG4-glycopeptides among HC, PDAC, and AIP groups. * represents *p<0.05;* ** represents *p <0.01;* *** represents *p <0.0001 for Mann-Whitney statistical analysis.*

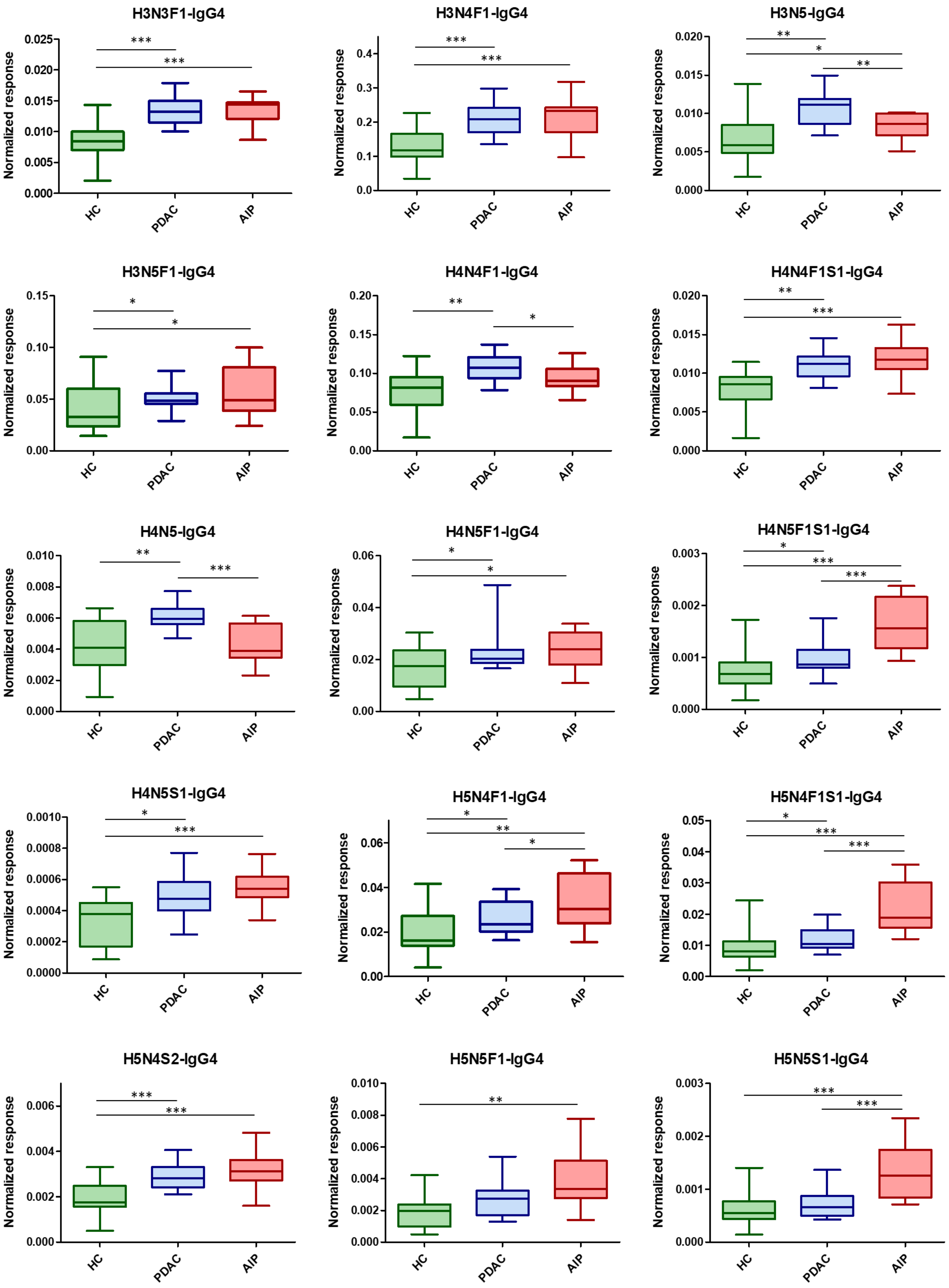

Supplement: Supplementary file 1 [file ijms-22-11527-s001.zip › Supplementary Figure S2_15 IgG4 glycopeptides.pdf]
