# Supplementary material for: Affinity Purification Coupled to Stable Isotope Dilution LC-MS/MS Analysis to Discover IgG4 Glycosylation Profiles for Autoimmune Pancreatitis
Source: Int J Mol Sci. 2021 Oct 26;22(21):11527. doi: 10.3390/ijms222111527 (PMC8584136; doi:10.3390/ijms222111527)
Supplement: Supplementary file 1 [file ijms-22-11527-s001.zip › Supplementary Table S1_precision and accuracy.pdf]

Supplementary Table S1. Accuracy and precision evaluation of analytes at LLOQ, low, medium,and high QC samples.

Analytes	Spiked IgG4 Concentration ($\mu g\ \mu L^{-1}$)	RT Precision (RSD, %)	Intra-batch (n=5)		Inter-batch (three occasions)	
			Precision (RSD, %)	Accuracy (% Recovery)	Precision (RSD, %)	Accuracy (% Recovery)
IgG4	0.14	0.10	2.6	97.3±2.8	2.9	97.9±3.1
	0.41		2.0	100.0±2.1	2.5	101.5±2.6
	3.30		3.4	100.9±3.4	2.2	101.2±2.2
	6.60		1.2	96.8±1.2	1.4	97.1±1.4
H3N3F1-IgG4	0.14	0.35	20.3	100.5±22.7	36.4	89.9±29.4
	0.41		33.5	105.40±32.9	21.8	112.0±23.2
	3.30		8.2	93.0±7.7	10.8	92.0±9.8
	6.60		8.4	80.1±6.5	7.8	83.0±6.5
H3N4F1-IgG4	0.14	0.12	4.9	81.4±5.7	7.9	82.7±9.3
	0.41		5.8	100.4±6.6	7.3	101.8±8.3
	3.30		3.0	88.2±2.7	4.0	90.3±3.7
	6.60		2.8	80.9±2.3	3.2	81.5±2.6
H3N5-IgG4[a]	0.14	0.25	NA	NA	NA	NA
	0.41		17.7	86.3±20.8	22.0	106.2±30.0
	3.30		7.4	80.4±6.2	15.7	89.4±14.7
	6.60		6.4	72.3±4.7	12.6	77.9±10.1
H3N5F1-IgG4	0.14	0.09	18.2	87.4±20.0	12.4	88.9±13.2
	0.41		5.6	100.1±6.4	9.3	100.9±9.9
	3.30		3.5	88.3±3.1	5.8	89.7±5.3
	6.60		2.5	80.7±20	4.5	80.7±3.6
H4N4F1-IgG4	0.14	0.10	4.3	89.7±4.9	6.1	88.0±7.0
	0.41		3.2	99.5±3.5	7.1	101.3±7.9
	3.30		3.8	87.9±3.4	5.4	89.5±4.9
	6.60		1.0	79.9±0.9	3.8	81.1±3.1
H4N4F1S1-IgG4	0.14	0.09	7.4	105.1±5.8	23.1	93.4±21.4
	0.41		13.3	97.2±14.0	12.3	98.6±12.4
	3.30		2.7	85.7±2.4	7.1	88.6±6.2
	6.60		4.5	81.3±3.7	4.5	82.1±3.7
H4N5-IgG4[a]	0.14	0.34	NA	NA	NA	NA

	0.41		9.1	93.3±12.0	22.0	105.2±28.0
	3.30		8.2	78.8±6.5	15.6	88.5±14.1
	6.60		3.8	72.9±3.0	11.4	76.9±8.9
H4N5F1-IgG4	0.14	0.20	13.9	87.4±12.7	16.4	90.2±16.8
	0.41		13.4	93.7±12.4	12.9	98.1±13.1
	3.30		4.2	86.7±3.7	6.8	88.3±6.1
	6.60		4.3	82.1±3.6	3.6	82.2±3.0
H4N5F1S1-IgG4[a]	0.14	0.21	NA	NA	NA	NA
	0.41		NA	NA	NA	NA
	3.30		16.1	87.9±14.1	13.9	88.9±13.2
	6.60		13.7	91.5±11.6	9.0	88.4±7.2
H4N5S1-IgG4[a]	0.14	0.57	NA	NA	NA	NA
	0.41		NA	NA	NA	NA
	3.30		19.4	74.8±13.7	25.4	94.6±23.5
	6.60		15.2	88.2±10.5	18.7	88.3±15.0
H5N4F1-IgG4	0.14	0.14	17.3	92.4±17.4	21.7	91.3±21.4
	0.41		9.2	100.4±9.2	13.0	102.7±13.6
	3.30		2.8	88.3±2.4	6.0	89.4±5.4
	6.60		3.7	82.2±3.0	3.6	82.2±2.9
H5N4F1S1-IgG4[a]	0.14	0.10	NA	NA	NA	NA
	0.41		17.3	88.4±14.0	19.4	96.3±17.7
	3.30		2.3	88.8±1.9	6.0	90.7±5.4
	6.60		1.8	83.8±1.6	4.4	83.4±3.7
H5N4S2-IgG4[a]	0.14	0.26	NA	NA	NA	NA
	0.41		NA	NA	NA	NA
	3.30		15.3	83.6±14.4	19.1	89.3±17.4
	6.60		13.7	82.1±11.1	10.5	82.0±8.3
H5N5F1-IgG4[a]	0.14	0.25	NA	NA	NA	NA
	0.41		NA	NA	NA	NA
	3.30		7.1	92.6±6.2	6.8	91.1±6.0
	6.60		5.7	84.2±4.8	6.3	82.1±5.4
H5N5S1-IgG4[a]	0.14	0.40	NA	NA	NA	NA
	0.41		NA	NA	NA	NA
	3.30		26.1	79.5±15.4	28.5	95.1±23.5

6.60		12.3	80.9±10.9	20.0	83.2±17.5

a Analytes with smaller signals which the precision and accuracy results were not available from LLOQ or low QC samples.
