# Supplementary material for: Affinity Purification Coupled to Stable Isotope Dilution LC-MS/MS Analysis to Discover IgG4 Glycosylation Profiles for Autoimmune Pancreatitis
Source: Int J Mol Sci. 2021 Oct 26;22(21):11527. doi: 10.3390/ijms222111527 (PMC8584136; doi:10.3390/ijms222111527)
Supplement: Supplementary file 1 [file ijms-22-11527-s001.zip › Supplementary Table S2_stabilities.pdf]

Supplementary Table S2. Long-term stability, freeze-thaw cycles stability, and post-processstability of IgG4 samples kept at -20 ℃ freezer or autosampler at specific conditions.

IgG4	Stability			
	7 days (%)	15 days (%)	freeze-thaw[c] (%)	Autosampler stability[d] (post-process, %)
Low[a,b]	100.8 ± 1.2	98.2 ± 0.7	92.1 ± 0.9	96.0 ± 8.5
Medium	104.1 ± 1.1	99.1 ± 0.8	85.3 ± 1.4	89.4 ± 3.1
High	100.8 ± 1.9	100.1 ± 0.6	93.7 ± 0.4	92.2 ± 1.7

[a] The spiked concentrations of human IgG4 were 0.41, 3.3, and 6.6 μg μL^{-1} for low, medium, and high QC samples.

[b] The results were performed as: average ± standard deviation

[c] Three freeze-thaw cycles were conducted for evaluation.

[d] The samples were kept in 6℃ autosampler (24 hrs) for evaluation.
